# Evaluation of Meibomian Gland Function after Therapy of Eyelid Tumors at Palpebral Margin with Super Pulse CO_2_ Laser

**DOI:** 10.1155/2022/8705436

**Published:** 2022-01-17

**Authors:** Songjiao Zhao, Jueni Duan, Jing Zhang, Lan Gong

**Affiliations:** ^1^Department of Ophthalmology, Eye, Ear, Nose, & Throat Hospital of Fudan University, Shanghai 200031, China; ^2^Department of Physical Examination, Shenzhen University General Hospital, China; ^3^Department of Facial Plastic and Reconstructive Surgery, Eye, Ear, Nose, & Throat Hospital of Fudan University, Shanghai 200031, China

## Abstract

**Purpose:**

To investigate the effect on meibomian gland function of super pulse carbon dioxide (CO_2_) laser excision in the treatment of eyelid tumors at palpebral margin.

**Methods:**

36 patients with 36 eyelid tumor size ≤ 1 cm and within 1 mm to palpebral margin were recruited in this study. Of which, 16 cases with tumors in the upper eyelid and 20 cases in the lower eyelid were involved. The eyelid tumors of all the patients were treated by super pulse CO_2_ laser with its power density varied between 0.6 and 21.1 W/mm^2^ and in repeat mode. The laser spot size ranged from 120 to 200 *μ*m. Ocular surface parameters including tear film break-up time (BUT) and meibograde, meibum expressibility, and meibum quality were evaluated at pretherapy, 1 week, 1 month, and 3 months posttherapy in all 36 patients.

**Result:**

All the patients were satisfied with the therapy. No infective complications and recurrence occurred in any of the 36 patients at the following period. The eyelid wound recovered well with nearly normal appearing after 2 to 3 weeks. The morphology of limbi palpebralis, BUT, meibograde, meibum expressibility, and meibum quality of all the 36 patients showed no significant difference before and after the therapy.

**Conclusions:**

Super pulse CO_2_ laser had no effect on meibomian gland function and morphology in the excision of tumors at palpebral margins, which was an efficacy and well-tolerated therapy with lower complications and recurrence.

## 1. Introduction

Eyelid benign tumors are common cosmetic concerns and traditionally treated by surgical excision in daily ophthalmology practice. However, specific surgical difficulties and complications such as eyelid deformity and scar formation following surgery should be considered in tumors located at palpebral margin. Laser therapy, for instance, carbon dioxide with infrared source (CO_2_, 10600 nm) [1], erbium yttrium aluminum garnet laser (Er:YAG, 2790–2940 nm) [2], and argon laser (Ar, 488–514 nm) [3] has been widely used and demonstrated superior to traditional surgery in the treatment of eyelid tumor for the shorter operation time, less pain, no bleeding, mild postoperative reaction, no scar, satisfactory healing result, and good acceptable by patients. The application of CO_2_ laser in a super pulse model has been proved effectively and predominantly in the treatment of eyelid benign tumors for the shortened exposure time and reduced thermal damage [1, 4–6].

Meibomian gland is a critical sebaceous gland that is located in the superior and inferior tarsal plates and opened at the palpebral margin. The meibum is delivered from the opening to the ocular surface to maintain the tear film stability and ocular surface homeostasis [7]. Hyposecretion or obstruction would affect meibomian gland function, even leading to meibomian gland dysfunction. Several iatrogenic conditions such as eyelid surgery and cosmetic procedures were identified and reduced the outflow of lipid secretion because of inadequate gland squeezing, inflammatory obstruction of the opening, and even complete loss of meibomian gland [8–10]. Although super pulse CO_2_ laser has been demonstrated superior to other traditional lasers, it has been reported to form atrophic scarring at the treated lesion [4]. Furthermore, the safety and efficacy of super pulse CO_2_ laser in therapy of tumor at palpebral margin has not been investigated. Considering that eyelid tumor at palpebral margin is generally colocalized with the opening of meibomian gland, we wondered whether the inflammation or scar formation of palpebral margin following super pulse CO_2_ laser excision would have effect on meibomian gland function.

In this study, we investigated the effect of super pulse CO_2_ laser excision on meibomian gland function in the treatment of eyelid tumors at palpebral margin. The safety and cosmetic effect of super pulse CO_2_ laser was also evaluated.

## 2. Methods

### 2.1. Patients

Thirty-six patients who underwent super pulse CO_2_ laser-assisted benign eyelid tumor excision at the Eye, Ear, Nose and Throat Hospital of Fudan University, from May 2020 to March 2021, were recruited in this study. Written informed consent was obtained from all the participants. The exclusion criteria included premalignant lesions, large lesions (diameter > 1 cm), and blepharitis. The study conformed to the Declaration of Helsinki and was approved by the ethics committee of the Eye, Ear, Nose, and Throat Hospital of Fudan University (2016034).

### 2.2. Data Collection and Ocular Examination

General ophthalmologic examination including visual acuity, intraocular pressure, anterior segment and fundus, and eyelid examination was performed before treatment. Age, gender, operated eye and eyelid, the number of lesions, and the size of lesions were collected. All the patients received ocular surface examination including tear film break-up time (BUT), meibograde, and meibomian gland function before treatment and 1 week, 1 month, and 3 months after treatment. Intraoperative and postoperative complications or recurrence were also recorded.

### 2.3. Super Pulse CO_2_ Laser-Assisted Eyelid Tumor Excision

**Figure 1:**
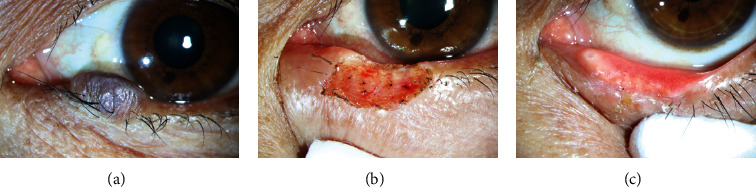
Pigmented nevus located at palpebral margin and close to the papilla: (a) before the laser therapy, (b) just after the laser therapy, and (c) 1 month after the laser therapy.

All the patients underwent super pulse CO_2_ laser-assisted eyelid tumor excision by the same senior oculoplastic surgeon (Jing Zhang) under topical anesthesia with 2% lidocaine. A corneal protection plate was used to prevent ocular injury. The lesion basement was excised using a CO_2_ laser (wave length: 10600 nm; power density: 0.6-21.1 W/mm^2^; exposure time: 0.05 seconds) with the help of a biopsy forceps. Then, a larger spot of CO_2_ laser (150-200 *μ*m) was used to photocoagulate the margin and bottom of the treated area to form a shallow pit. Histopathological examination was performed for the resected tumors with sufficient residual tissue. Topical chlortetracycline ointment was used immediately after the treatment. Patients were informed to keep the treated area dry until the crust desquamated and fell off.  Figure 1 shows the picture of pigmented nevus before and after super pulse CO_2_ laser treatment.

### 2.4. Tear Film Break-Up Time (BUT)

A minimal volume fluorescein was instilled into the lower fornix of patients using the fluorescein strips (Jingming, Tianjing, China). The patients were requested to blink several times naturally and then look straight and keep eyes open, until the first dry spots on the cornea surface appeared under the cobalt blue light of the slit lamp. The time (seconds) from the last blink to the first appearance of dry spots was recorded as BUT. This procedure was repeated for three times.

### 2.5. Meibograde

**Figure 2:**
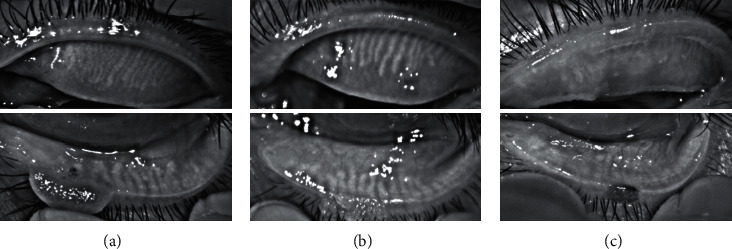
Meibographies of the upper and lower eyelids showing different meibomian gland dropouts: (a) less than one third at both upper and lower eyelids, (b) between one third and two thirds at both upper and lower eyelids, and (c) over two thirds at both upper and lower eyelids.

Meibographies of the upper and lower eyelids were captured by the Oculus Keratograph 5 M (Wetzlar, Germany), and the meibomian gland dropout rate was analyzed qualitatively by ImageJ software (National Institutes of Health, USA). Meibograde of each eyelid was graded based on the meibomian gland dropout as 0-3: 0, no dropout of meibomian gland; 1, dropout was less than one third of the meibomian gland; 2, dropout was between one third and two thirds of the meibomian gland; and 3, dropout was over two thirds of the meibomian gland. Meibogrades of the upper and lower eyelid were summed to grade as 0-6 for each eye (Figure 2).

### 2.6. Meibomian Gland Function

The assessment of meibomian gland function was under slitlamp using a grade method considering meibomian gland expressibility, meibum quality, and lid margin changes. Over 8 meibomian glands of laser treated eyelid were digitally pressed, and the meibum expressibility was graded as 0-3: 1, light; 2, moderate; and 3, heavy pressure, as well as the quality of meibum was graded as 0-3: 0, clear; 1, cloudy; 2, granular; and 3, toothpaste. Based on the irregularity of the lid margin, telangiectasia, plugging of meibomian orifices, and replacement of the mucocutaneous junction, lid margin changes were graded as 0-4.

### 2.7. Statistical Analyses

Data analyses were performed using Graphpad Prism 9 software (USA). The results are expressed as the mean ± standard deviation. Chi square test was used to compare BUT, meibograde, meibum expressibility, and meibum quality before and after treatment.

## 3. Results

A total of 36 patients (36 eyes with 36 eyelid tumors) that underwent super pulse CO_2_ laser-assisted eyelid tumor excision were included, with 11 males and 25 females. The mean age at the time of therapy was 38.1 ± 15.3 years (range from 11 years to 65 years). The tumors were primarily located at the left eye (*n* = 20, 55.6%), and the lower eyelid was more involved (*n* = 20, 55.6%). The lesion size was 1 cm at most, including 24 lesions ≤ 5 mm and 13 lesions > 5 mm. Before the CO_2_ laser treatment, the mean values of BUT, meibograde, meibum expressibility, and meibum quality in the treated eyes were 5.4 ± 2.8 s, 2.9 ± 1.0, 2.9 ± 1.0, and 1.6 ± 0.9, respectively (Table 1).

The wound healing was satisfactory in all the 36 patients without any infection. None of the patients were complicated by lid notching, entropion, or ectropion. Eyelash loss was occurred in 4 patients (11.1%) with lesions larger than 5 mm. Transient hypopigmentation was occurred in 3 patients (8.3%). No distinctive hyperpigmentation, scar hypertrophy, blistering, or swelling at the treated areas. No systematic complications or recurrence was observed in the following period or recurrence.

Histopathological diagnosis was clarified in 32 patients (88.9%). Pigmented nevus (*n* = 13, 40.6%) and squamous papilloma (*n* = 11, 34.4%) were the two primary types, followed by intradermal nevus (*n* = 4, 12.5%), xanthelasma palpebrarum (*n* = 2, 6.3%), viral wart (*n* = 1, 3.1%), and dermis collagen fiber hyperplasia (*n* = 1, 3.1%) (Table 2).

**Figure 3:**
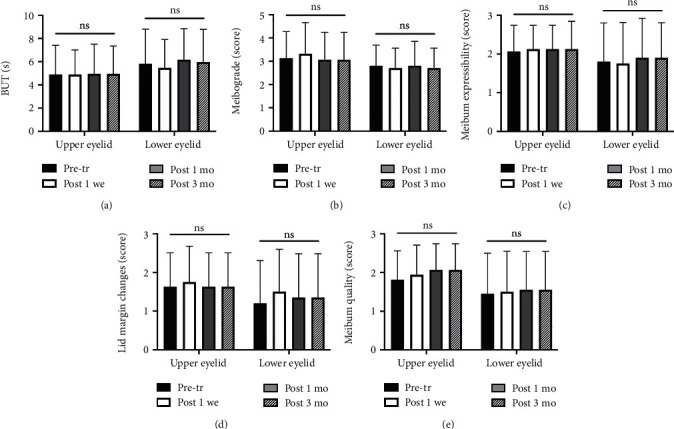
Changes in tear film parameter and meibomian gland function after super pulse CO_2_ laser therapy. Evaluation of the BUT (a), meibograde (b), meibum expressibility (c), lid margin changes (d), and meibum quality (e) at pretherapy and posttherapy in the eyes with upper or lower eyelid tumors. No significant differences were found at any follow-up time compared to the pretreated values. The super pulse CO_2_ laser therapy showed no significant difference between upper and lower eyelid tumors.

After the CO_2_ laser treatment, BUT did not significantly changed in eyes with upper or lower eyelid tumors (Figure 3(a)). Compared with the meibograde and meibum expressibility from the pretreated values, no significant difference was found at any follow-up time in eyes with upper or lower eyelid tumors (Figures 3(b) and 3(c)). The lid margin changes showed an insignificant increase at 1 week posttherapy and returned to the basal level at 1 month posttherapy in eyes with lower eyelid tumors (Figure 3(d)). Although the score of meibum quality gently increased after the treatment, the changing trend was not statistically significant in eyes with both upper and lower eyelid tumors (Figure 3(e)). The super pulse CO_2_ laser treatment in upper and lower eyelid tumors showed no significant difference.

## 4. Discussion

This study demonstrated that super pulse CO_2_ laser excision showed no significant effect on meibomian gland function in therapy of eyelid benign lesions at palpebral margin, serving as a safe and effective alternative therapeutic modality of traditional surgical excision.

Eyelid benign tumors accounted for nearly 80% of eyelid tumors, and not a few proportion was existed at palpebral margins in different types of pathology [11]. The palpebral margin is a complex structure with eyelash follicles and exocrine gland located such as meibomian gland and sebaceous gland. Traditional surgical excision of tumors at palpebral margins was a challenge because of the complications such as scar hypertrophy, eyelash loss, and angulation deformity, leading to patients' dissatisfaction.

Advancement of laser technology in recent two decades has increased options for eyelid benign tumor treatment, which achieved precise tissue damage by using appropriate wavelength and pulse duration of laser energy [12]. Comparing with other laser therapy, super pulse CO_2_ lasers enables hemorrhage-free noncontact incisional excision and that producing superior tissue contraction and hemostasis, especially in the deeper lesion removal [2]. For tumors with special location such as palpebral margins, CO_2_ laser surgery keeps the margins in their anatomical position so that entropion or ectropion is not to be reckoned with, showing advantageous in preserving patients' appearance with complete morphology of palpebral margin [1, 5, 13].

Although CO_2_ laser therapy exhibits more thermal necrosis and slower wound healing times that would be accompanied by the longer inflammatory reaction [12], the satisfactory therapeutic and cosmetic results with reduced complications and recurrence rate were confirmed by numerous studies in eyelid benign lesions [1, 4, 5, 14–17].

Consistent with our previous reports, this study also identified the good clinical response of super pulse CO_2_ laser in excision of eyelid benign tumors without any recurrence in the follow-up period [1, 13]. Here, the original contribution was to further investigate the potential effects of super pulse CO_2_ laser on the meibomian gland. Meibomian glands are distributed on the posterior edged of the palpebral margin, participating in the ocular surface homeostasis [7]. Persistent inflammation of eyelid margin or ocular surface might lead to the infiltration of inflammatory cells in the surrounding microenvironment that serves as an inflammatory form of meibomian gland dysfunction [18]. Ocular surgeries such as cosmetic blepharoplasty and cataract have been demonstrated to effect on meibomian gland function owing to direct damage or ocular surface inflammation [19, 20]. In the super pulse CO_2_ laser therapy of eyelid benign tumors at palpebral margin, the intensive heat generated by CO_2_ laser directly destroys the surrounding tissue of the lesions. The local and adjacent meibomian gland might be potentially affected due to the inflammatory cells infiltration or thermal injury of the opening at the margin, which at risk of hyperkeratinization of resulting in degenerative gland dilatation and atrophy [21]. Based on this, we investigate the changes of meibomian gland morphology and function after the super pulse CO_2_ laser excision. To our amazement, except for those preoperative atrophy, neither meibomian gland function nor morphology was affected by the super pulse CO_2_ laser in the following period. Furthermore, the tear film stability remains unchanged after the procedure. This might be contributed to the short pulse duration (microseconds) and narrower zone of coagulation of super pulse CO_2_ laser. Comparing with traditional CO_2_ laser, super pulse CO_2_ laser provides a sufficient interval time to cool the surrounding tissue, limiting thermal damage and inflammatory infiltration [1, 22], which might have less thermal damage and functional effect of meibomian gland. To the best of our knowledge, this is the first study that identified the nonsignificant effect of super pulse CO_2_ laser on meibomian gland.

There are several limitations of this study including the relative small subject number and follow-up period (3 months). Besides, no control group treated with traditional laser therapy or surgical excision was included in this study. Further study should be conducted with a longer follow-up time and a larger subject number, comparing with traditional treatment group, to make a more convincing report.

## 5. Conclusion

Our study identified that super pulse CO_2_ laser had no effect on meibomian gland function and morphology in the excision of tumors at palpebral margins, which was an efficacy and well-tolerated therapy with lower complications and recurrence.

## Figures and Tables

**Table 1:** Demographic and preoperative characteristics.

Characteristics	*n* = 36
Age (mean ± SD)	38.1 ± 15.3
Sex, *n* (%)	
Male	11 (30.6%)
Female	25 (69.4%)
Laterality, *n* (%)	
Left	20 (55.6%)
Right	16 (44.4%)
Eyelid, *n* (%)	
Upper	16 (44.4%)
Lower	20 (55.6%)
Size of lesions, *n* (%)	
≤5 mm	24 (64.8%)
5-10 mm	13 (35.2)
BUT (s, mean ± SD)	5.4 ± 2.8
Meibograde (mean ± SD)	2.9 ± 1.0
Meibum expressibility (mean ± SD)	1.9 ± 0.9
Meibum quality (mean ± SD)	1.6 ± 0.9
BUT: tear film break-up time	

**Table 2:** The histopathological diagnosis of the benign eyelid lesions treated with CO_2_ laser.

Histopathological diagnosis	*N* = 32 (100%)
Pigmented nevus	13 (40.6%)
Squamous papilloma	11 (34.4%)
Intradermal nevus	4 (12.5%)
Xanthelasma palpebrarum	2 (6.3%)
Viral wart	1 (3.1%)
Dermis collagen fiber hyperplasia	1 (3.1%)
Histopathological diagnosis was clarified in 32/36 patients (88.9%).	

## Data Availability

The datasets used and/or analyzed during the current study are available from the corresponding author on reasonable request.
